# Pulmonary nodules in Denmark: occurrence, resource use, and risk of lung cancer and death

**DOI:** 10.2340/1651-226X.2025.44711

**Published:** 2025-11-02

**Authors:** Cecilia Hvitfeldt Fuglsang, Ina Trolle Andersen, Frederik Pagh Bredahl Kristensen, Henriette Engberg, Morten Borg, Ole Hilberg, Torben Riis Rasmussen

**Affiliations:** a Department of Clinical Epidemiology, Aarhus University and Aarhus University Hospital, Aarhus, Denmark; b The Danish Health Care Quality Institute (DHQI), Odense, Denmark; c Department of Internal Medicine, Lillebaelt Hospital Vejle, Vejle, Denmark; d Department of Regional Health Research, Southern Danish University, Odense, Denmark; e Institute of Clinical Medicine, Aarhus University, Aarhus, Denmark; f Department of Respiratory Diseases and Allergy, Aarhus University Hospital, Aarhus, Denmark; g The Danish Lung Cancer Registry, Odense University Hospital, Odense, Denmark

**Keywords:** Pulmonary nodules, epidemiology, lung cancer, chest CT scans, mortality

## Abstract

**Background and purpose:**

Little is known about how pulmonary nodules are managed in routine clinical care. We examined their occurrence, the use of computed tomography (CT) scans, referrals to cancer pathways, and the risk of lung cancer and death post-diagnosis.

**Patients/material and methods:**

We conducted a population-based cohort study using Danish health registry data. We identified all adults with a first-time pulmonary nodule diagnosis from 2018 to 2022. We examined the incidence of pulmonary nodules using age- and sex-standardized incidence rates (SIRs). We used the Aalen-Johansen estimator to calculate the probability of receiving a chest CT scan, a cancer patient pathway referral, the risk of lung cancer, and mortality within 12 months after a nodule diagnosis.

**Results:**

We identified 43,209 patients with a pulmonary nodule diagnosis. The age- and sex-SIR of pulmonary nodules was 197 per 100,000 person-years in 2018, declining to 186 per 100,000 person-years in 2022. Within 12 months after a nodule diagnosis, 68.3% of the cohort underwent at least one chest CT scan, with 51.0% receiving a low-dose chest CT scan and 7.2% receiving a referral to a lung cancer patient pathway. The 12-month lung cancer risk was 3.6% (95% CI, 3.4 to 3.8%), with the highest risk for stage I lung cancer, and the mortality was 7.0% (95% CI, 6.8 to 7.3%).

**Interpretation:**

The incidence of pulmonary nodules remained relatively stable from 2018 to 2022. More than 30% of patients with nodules lacked a chest CT scan within 12 months after a pulmonary nodule diagnosis.

## Introduction

Cancers of the trachea, bronchi, and lungs are the leading cause of cancer deaths in the world [1]. These cancers are often diagnosed at advanced stages, contributing to the high mortality [1]. In Denmark, only one out of three lung cancer patients is diagnosed at a resectable stage [2].

Pulmonary nodules are frequently detected, with a prevalence of up to 50% in high-risk populations [3–5]. Although most nodules are benign, up to 4% in high-risk patients are early-stage lung cancer [6]. Routine imaging often cannot distinguish between benign and malignant nodules, and invasive procedures are impractical for nodules below 8 mm, limiting accurate classification [5]. Therefore, patients with pulmonary nodules often undergo serial low-dose computed tomography (CT) scans to exclude malignancy, with follow-up intervals determined by individual risk factors and nodule characteristics [3–5, 7]. While this approach facilitates early detection of lung cancers, it also exposes patients to repeated radiation, may cause unnecessary anxiety, and requires substantial healthcare resources.

Over the past decade, the increasing number of CT scans has led to a higher incidence of incidentally detected pulmonary nodules and a substantial allocation of resources to nodule follow-up programs [4, 5, 8, 9]. Previous studies have found that 29 to 58% of patients with a pulmonary nodule attended a follow-up CT scan after a nodule diagnosis [4, 10–14]. However, these studies have been relatively small (~1,100 patients or fewer) and focused on selected populations (e.g. veterans [13] or emergency department patients undergoing imaging for suspected pulmonary embolus [10]). Consequently, the occurrence of pulmonary nodules in the general population and their management in routine clinical care remain unclear [7].

To address these evidence gaps, we used population-based Danish health registers to conduct a cohort study. We investigated (1) the occurrence of pulmonary nodules; (2) resource utilization in terms of chest CT scans and referrals to cancer patient pathways, and (3) the risk of lung cancer and death after pulmonary nodule diagnosis.

## Patients/materials and methods

### Study design, registries, and setting

We conducted a population-based cohort study in Denmark using individual-level registry data from 2018 to 2022. This study was reported according to the STROBE guidelines. According to Danish legislation, informed consent and approval from an ethics committee are not required for registry-based studies.

The Danish healthcare system is tax-funded and provides free access to primary and secondary healthcare for all Danish residents [15]. All residents of Denmark receive a unique personal identifier at birth or upon immigration, enabling individual-level linkage to registry data [16]. We used the following registries in the study. The Danish Civil Registration System (CRS) has existed since 1968 and records daily updates on vital and immigration status [16]. The Danish National Patient Registry (DNPR) has recorded all inpatient hospital contacts since 1978 and all outpatient and emergency department contacts since 1995. Reporting to the DNPR from private hospitals and outpatient clinics has been compulsory in Denmark since 2003 [17]. The DNPR records all pulmonary nodules regardless of size based on discharge diagnosis codes. The Danish Cancer Registry (DCR) has recorded all incident cancers from 1943 and forth, with detailed information on cancer diagnosis and tumor stage according to the TNM-classification [18].

Pulmonary nodules in Denmark are followed up according to the British Thoracic Society guidelines for the investigation and management of pulmonary nodules (2015) and/or the Guidelines for the Management of Incidental Pulmonary Nodules Detected on CT Images (the Fleischner Society 2017) [3, 19]. These guidelines for single pulmonary nodules recommend a low-dose radiation chest CT scan without administration of contrast medium (low-dose chest CT scan) at 3 months for large nodules ≥ 8 mm, while nodules < 8 mm should be monitored with scans at 6–12 months and again at 12, 18, or 24 months, depending on nodule size and individual risk factors [3, 19]. If malignancy is suspected, the person is referred to further diagnostic evaluation through a standardized cancer patient pathway, where other CT modalities are used, including contrast-enhanced CT scans and positron emission tomography/CT scans. The cancer patient pathways were introduced in 2008 to obtain a timely diagnosis and improved prognosis for cancer patients [20].

### Study population

**Figure 1:**
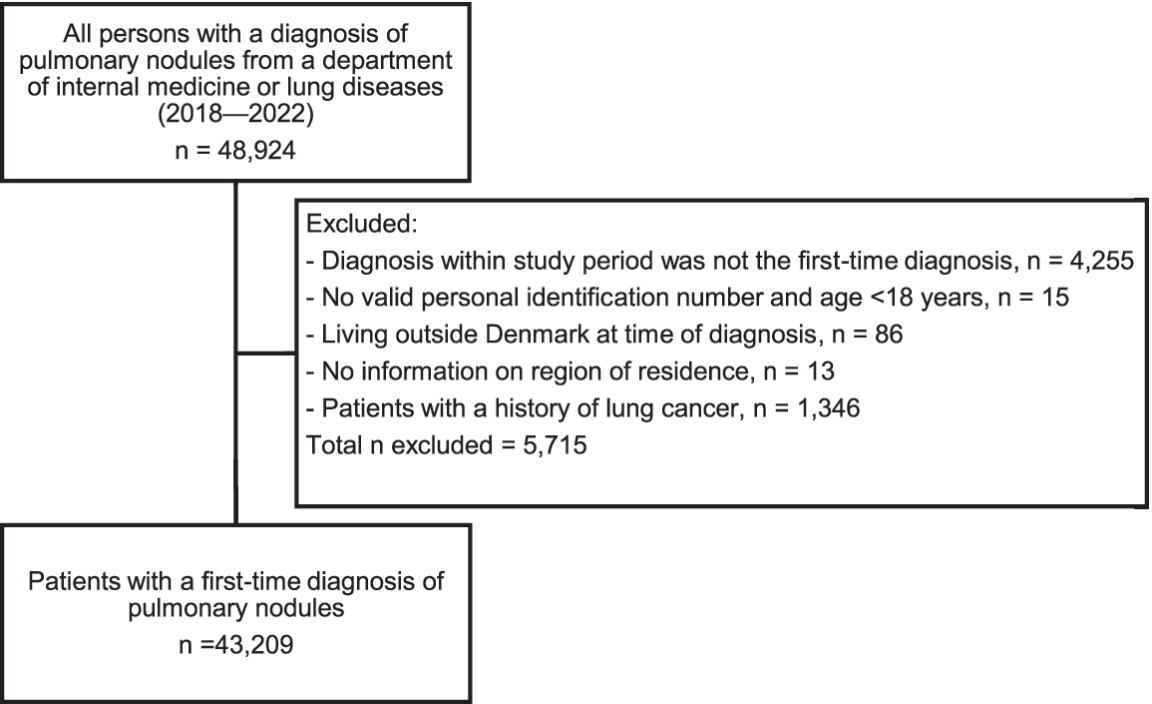
Cohort sampling.

We identified all patients in Denmark aged ≥18 years with a first-time diagnosis of a pulmonary nodule during the study period. The date of hospital admission or first outpatient visit was the index date. Patients with nodules were identified using the diagnosis code for ‘abnormal imaging of the lungs’ (R91.9). While this code primarily applies to pulmonary nodules, it may include larger infiltrates requiring follow-up CT scans. Based on input from clinicians in several respiratory disease departments, we increased the specificity by including only patients diagnosed in internal or respiratory disease departments, as nodules detected in other hospital departments are often referred to these specialties for follow-up. Our a priori analysis confirmed that over 90% of all patients with a discharge diagnosis of R91.9 were treated in an internal or respiratory department. Patients with a prior lung cancer diagnosis recorded in DCR were excluded (Figure 1).

### Outcomes

We examined the occurrence of pulmonary nodules, resource utilization as part of the pulmonary nodules follow-up program, and the risk of lung cancer and mortality. The data were ascertained from the DNPR and DCR, and the exact date of death was ascertained from the CRS. We examined the incidence and prevalence of pulmonary nodules in the Danish population during the study period. To estimate resource use, we computed the probability and average number of receiving any hospital chest CT scan, a low-dose chest CT scan used in the pulmonary nodule follow-up program, and referral to cancer patient pathways after a pulmonary nodule diagnosis. We examined the risk of incident lung cancer after a pulmonary nodule diagnosis overall and according to the stage. The positive predictive value of lung cancer diagnosis and stage is above 90% in the Danish registries [21].

### Covariates

We obtained information on age, sex, and region of residence from the CRS. We obtained data from the DCR on cancers other than lung cancer and from the DNPR regarding hospital departments where the nodule was diagnosed, the hospital history of comorbidities, including chronic obstructive pulmonary disease (COPD), and the history of referrals to cancer patient pathways. The cancer patient pathway referral could either be active, requiring further diagnostic tests, or closed, indicating the completion of diagnostic cancer tests. Codes and definitions for all covariates are available in Supplemental Table 1.

### Statistical analyses

#### Occurrence

We calculated crude and age- and sex-standardized incidence rates (SIRs) of pulmonary nodules per 100,000 person-years for each year in the study period by direct standardization using the Danish population of January 1, 2018, as the reference population. The prevalence of pulmonary nodules was calculated for patients alive on January 1, 2023, identifying all previous nodule diagnoses. As the incidence may be influenced by the increasing number of CT scans over time [4], we calculated the number of any chest CT scans and the number of patients who received a chest CT scan for each year during the study period.

#### Resource use

We presented characteristics of patients with pulmonary nodules. We followed the patients from the date of diagnosis until the date of a chest CT scan, date of lung cancer diagnosis, death, emigration, 30 months after the index date, or end of follow-up (December 31, 2022), whichever occurred first. We used the Aalen-Johansen estimator to calculate the cumulative incidence (probability) of receiving any chest CT scan and a low-dose chest CT scan. We considered death and incident lung cancer diagnosis as competing events, as this analysis focused on resource use in the pulmonary nodule follow-up program rather than outcomes related to lung cancer. We used mean cumulative count (MCC) to calculate the average number of chest CT scans per 100 persons, again treating death and lung cancer as competing events [22]. The outcome probabilities and MCCs were reported at months 3, 6, 12, 18, and 24 after the pulmonary diagnosis, as per the follow-up scheme outlined in the clinical guidelines (Supplementary Figure 2A for study design illustration) [23]. A similar approach was employed to calculate the probability of referral to cancer patient pathways. All analyses were unadjusted, thus focusing on describing probabilities.

#### Risk of lung cancer and mortality

We followed patients from the date of pulmonary nodule diagnosis until the date of lung cancer diagnosis, death, emigration, 30 months after the index date, or the end of follow-up (December 31, 2022), whichever occurred first. We used the Aalen-Johansen estimator to calculate the risk of lung cancer, considering death a competing risk. We used the unadjusted Kaplan-Meier estimator to estimate all-cause mortality. Risks were reported at 6-, 12-, 18-, and 24-month follow-up times.

#### Additional analyses

We performed two additional analyses:

1. To assess lung cancer risk in patients with pulmonary nodules who did not have any follow-up chest CT scan, we conducted a landmark analysis at 12+1 months after the pulmonary nodule diagnosis. This landmark aligned with the longest follow-up interval in clinical guidelines, allowing all patients to receive one CT scan [3, 19]. The cohort included patients who had lung cancer-free survival (*n* = 29,869 [69%], Supplemental Figure 1), and we compared incident lung cancer risks between those who had and had not received a chest CT scan during the period.
2. We sampled a cohort of patients with incident lung cancer during 2018 to 2022 to assess the use of CT scans according to the presence of pulmonary nodules within 5 years preceding the lung cancer diagnosis (cohort sampling is described in the Supplemental Material and illustrated in Supplementary Figure 2B). We calculated the total number and average number of any chest CT scans, low-dose chest CT scans, and chest CT scans with contrast. These numbers were also calculated for subgroups by age, sex, diagnosis year, cancer stage, region in Denmark, cancer history, and COPD.

## Results

### Occurrence

**Figure 2:**
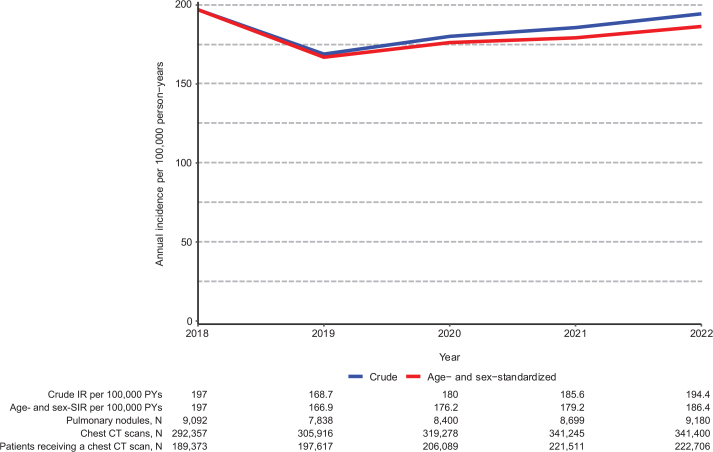
Annual incidence rates of pulmonary nodules between 2018 and 2022.

CT, chest tomography; PYs, person-years.

We identified 43,209 patients with a first-time pulmonary nodule diagnosis between 2018 and 2022 (Figure 1). We identified the lowest number of patients with nodules in 2019 (7,838 patients, 18.1%) and the largest in 2022 (9,180 patients, 21.2%). The total number of chest CT scans performed ranged from 292,257 scans in 2018 to 341,400 scans in 2022. The age- and sex-SIR of pulmonary nodules remained relatively stable with 197 per 100,000 person-years in 2018, gradually declining to 186 per 100,000 person-years in 2022 (Figure 2). As of January 1, 2023, the prevalence of diagnosed pulmonary nodules was 1,674 per 100,000 persons among Danish adults.

### Characteristics of patients with pulmonary nodules

Among the cohort, 53% were males, and the median age was 69 years (interquartile range: 59 years; 77 years). Among patients with a pulmonary nodule, 26.8% had a history of cancer, and 16.6% had a history of COPD. 33.1% were residing in the Capital Region, 26.9% in the Central Denmark Region, 15.5% in the Region Zealand, 15.4% in the Region of Southern Denmark, and 9.1% in the North Denmark Region. The majority (92.1%) were diagnosed at a department of pulmonary medicine (the diagnostic frequency by hospitals is presented in Supplemental Table 2). Within 6 months before the nodule diagnosis date, 32.9% had a previous referral diagnosis to a lung cancer patient pathway and 16.5% to another cancer patient pathway (Table 1).

**Table 1:** Descriptive characteristics of patients with pulmonary nodules from 2018 to 2022.

**Total, *n* (%)**	43,209 (100)
**Follow-up time in years (median, IQR)**	2.1 (0.9; 3.5)
**Sex, *n* (%)**	
Men	22,888 (53.0)
Women	20,321 (47.0)
**Age in years (median, IQR)**	69.2 (59.4; 76.5)
Age < 60	11,314 (26.2)
Age 60–70	11,309 (26.2)
Age 70–80	14,235 (32.9)
Age > 80	6,351 (14.7)
**Year, *n* (%)**	
2018	9,092 (21.0)
2019	7,838 (18.1)
2020	8,400 (19.4)
2021	8,699 (20.1)
2022	9,180 (21.2)
**Comorbidities, *n* (%)**	
COPD	7,181 (16.6)
Cancer (excl. lung cancer)	11,596 (26.8)
**Region of residence, *n* (%)**	
Capital Region of Denmark	14,288 (33.1)
Region Zealand	6,703 (15.5)
North Denmark Region	3,943 (9.1)
Central Denmark Region	11,611 (26.9)
Region of Southern Denmark	6,664 (15.4)
**Medical specialty, *n* (%)**	
Respiratory diseases	41,092 (92.2)
Internal medicine	3,463 (7.8)
**History of cancer patient pathway referral, *n* (%)**	
Lung cancer patient pathway at index date	1,734 (4.0)
Lung cancer patient pathway within 6 months before index date*	14,201 (32.9)
Other cancer patient pathway at index date	552 (1.3)
Other cancer patient pathway within 6 months prior to index date*	7,117 (16.5)

The index date was the date of diagnosis of the pulmonary nodule.

* Index date not included. COPD: chronic obstructive pulmonary disease.

### Resource use

**Figure 3:**
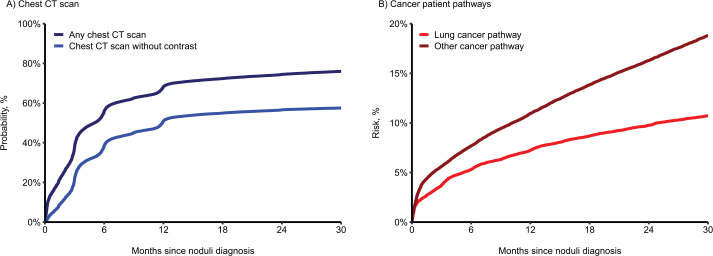
Resource use after a pulmonary nodule diagnosis. A) Probability of receiving a chest CT scan after a pulmonary nodule diagnosis; B) Risk of referral to a cancer patient pathway after a pulmonary nodule diagnosis.

The average number of chest CT scans was 192.1 per 100 individuals 24 months after pulmonary nodule diagnosis, totaling 83,004 CT scans among patients with nodules from 2018 to 2022 (Table 2). Within 6 months following a pulmonary nodule diagnosis, 56.1% had received at least one chest CT scan, and 38.8% had received at least one low-dose chest CT scan. After 12 months, the proportion increased to 68.3% for any chest CT scans and 51.0% for low-dose chest CT scans. At 24 months, the proportions were 74.4 and 56.6%, respectively (Table 2 and Figure 3).

**Table 2:** Cumulative incidence and mean cumulative counts of chest CT scan, lung cancer diagnosis, and death.

Outcome		Follow-up (months)	Cumulative incidence, % (95% CI)	Mean cumulative count of chest CT scans per 100 patients
**Any chest CT scan**		0–3	38.0 (37.5,38.5)	48.4
		0–6	56.1 (55.6,56.6)	83.8
		0–12	68.3 (67.8,68.8)	130.4
		0–18	72.3 (71.9,72.8)	164.3
		0–24	74.4 (74.0,74.9)	192.1
**Low-dose chest CT scan**		0–3	21.8 (21.4,22.2)	23.4
		0–6	38.8 (38.3,39.2)	45.9
		0–12	51.0 (50.5,51.5)	74.1
		0–18	54.9 (54.4,55.4)	93.1
		0–24	56.6 (56.1,57.1)	108.3
**Lung cancer patient pathway**		0–3	3.7 (3.5,3.9)	-
		0–6	5.3 (5.1,5.5)	-
		0–12	7.2 (7.0,7.5)	-
		0–18	8.7 (8.4,9.0)	-
		0–24	9.8 (9.5,10.1)	-
**Other cancer patient pathway**		0–3	5.6 (5.4,5.8)	-
		0–6	7.7 (7.4,8.0)	-
		0–12	11.0 (10.6,11.3)	-
		0–18	13.8 (13.5,14.2)	-
		0–24	16.3 (15.9,16.7)	-
**Lung cancer**	Overall	0–3	2.2 (2.1; 2.4)	-
	Stage I		0.6 (0.6, 0.7)	-
	Stage II		0.2 (0.2,0.3)	-
	Stage III		0.4 (0.4,0.5)	-
	Stage IV		0.8 (0.7,0.8)	-
	Stage unknown		0.2 (0.1, 0.2)	-
	Overall	0–6	2.8 (2.7,3.0)	-
	Stage I		0.9 (0.8,1.0)	-
	Stage II		0.3 (0.3,0.4)	-
	Stage III		0.5 (0.5,0.6)	-
	Stage IV		0.9 (0.8,1.0)	-
	Stage unknown		0.2 (0.2,0.2)	-
	Overall	0–12	3.6 (3.4,3.8)	-
	Stage I		1.4 (1.3,1.5)	-
	Stage II		0.4 (0.4,0.5)	-
	Stage III		0.6 (0.5,0.7)	-
	Stage IV		1.0 (0.9,1.1)	-
	Stage unknown		0.3 (0.2,0.3)	-
	Overall	0–18	4.3 (4.1,4.5)	-
	Stage I		1.7 (1.6,1.8)	-
	Stage II		0.5 (0.5,0.6)	-
	Stage III		0.7 (0.6,0.8)	-
	Stage IV		1.1 (1.0,1.2)	-
	Stage unknown		0.3 (0.3,0.4)	-
	Overall	0–24	4.7 (4.5,5.0)	-
	Stage I		1.9 (1.8,2.1)	-
	Stage II		0.6 (0.5,0.7)	-
	Stage III		0.8 (0.7,0.8)	-
	Stage IV		1.2 (1.1,1.3)	-
	Stage unknown		0.3 (0.3,0.4)	-
**Death**		0–3	2.4 (2.2,2.5)	-
		0–6	4.1 (3.9,4.3)	-
		0–12	7.0 (6.8,7.3)	-
		0–18	9.6 (9.3,9.9)	-
		0–24	12.0 (11.7,12.4)	-

COPD: chronic obstructive pulmonary disease; CT: computed tomography.

Within 6 months after a pulmonary nodule diagnosis, 5.3% of the cohort was referred to a lung cancer pathway and 7.7% to another type of cancer pathway. These numbers increased to 7.2 and 11.0% at 12 months, and 9.8 and 16.3% at 24 months (Table 2 and Figure 3).

### Risk of lung cancer and death

**Figure 4:**
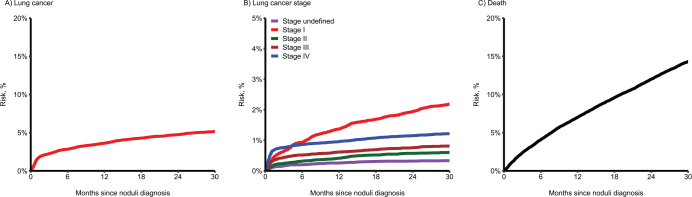
Risk of lung cancer and death following a pulmonary nodule diagnosis.

Among patients with a pulmonary nodule diagnosis, the risk of lung cancer was 2.8% (95% CI, 2.7 to 3.0%) at 6 months follow-up, 3.6% (95% CI, 3.4 to 3.8%) at 12 months, and 4.7% (95% CI, 4.5 to 5.0%) at 24 months (Table 2 and Figure 4). Patients with a pulmonary nodule were most likely diagnosed with either stage I or stage IV lung cancer (Figure 4). The mortality after a pulmonary nodule diagnosis was 4.1% (95% CI, 3.9 to 4.3%) at 6 months, 7.0% (95% CI, 6.8 to 7.3%) at 12 months, and 12.0% (95% CI, 11.7 to 12.4%) at 24 months follow-up (Table 2 and Figure 4).

### Additional analyses

Of the 29,869 patients with a pulmonary nodule diagnosis in the landmark analysis, 21,032 (70.4%) received at least one chest CT 12+1 months after the diagnosis (Supplemental Table 3). The risk of lung cancer at 11 months after the landmark (i.e. 24 months after nodule diagnosis) was 1.4% (95% CI, 1.3 to 1.6%) for patients who had received a chest CT scan and 0.4% (95% CI, 0.3 to 0.5%) for patients who had not received a chest CT scan in the period (Supplemental Table 4 and Supplemental Figure 3).

From 2018 to 2022, we identified 22,492 patients diagnosed with lung cancer, with 2,727 (12.1%) having a pulmonary nodule diagnosis within 5 years before the lung cancer diagnosis (Table 3). Patients with a pulmonary nodule diagnosis were more likely to have stage I lung cancer (41.8% vs. 18.9%) and less likely to have stage IV lung cancer (25.3% vs. 48.5%). Within 5 years before the lung cancer diagnosis, patients with a pulmonary nodule diagnosis were more likely to receive a chest CT scan (79.9% vs. 37.7%). The average number of chest CT scans per person per year was 0.52 for patients with nodules and 0.12 for patients without nodules. This number was consistent across subgroups (Supplemental Table 5).

**Table 3:** Characteristics of patients diagnosed with lung cancer from 2018 to 2022, according to a preceding pulmonary nodule diagnosis.

	All patients with lung cancer	No pulmonary nodule diagnosis within 5 years	A pulmonary nodule diagnosis within 5 years
**Number of patients**	22,492 (100.0)	19,765 (87.9)	2,727 (12.1)
**Women, *N* (%)**	11,609 (51.6)	10,149 (51.3)	1,460 (53.5)
**Age, median (Q1–Q3)**	72.6 (65.9;78.2)	72.5 (65.7;78.2)	73.5 (67.2;78.4)
**Age, *N* (%)**			
< 60	2,498 (11.1)	2,268 (11.5)	230 (8.4)
60–70	6,349 (28.2)	5,623 (28.4)	726 (26.6)
70–80	9,497 (42.2)	8,238 (41.7)	1,259 (46.2)
≥ 80	4,148 (18.4)	3,636 (18.4)	512 (18.8)
**Index year, *N* (%)**			
2018	4,353 (19.4)	3,862 (19.5)	491 (18.0)
2019	4,369 (19.4)	3,891 (19.7)	478 (17.5)
2020	4,570 (20.3)	3,981 (20.1)	589 (21.6)
2021	4,610 (20.5)	4,029 (20.4)	581 (21.3)
2022	4,590 (20.4)	4,002 (20.2)	588 (21.6)
**Region of residence, *N* (%)**			
North Denmark Region	3,035 (13.5)	2,762 (14.0)	273 (10.0)
Central Denmark Region	5,564 (24.7)	4,711 (23.8)	853 (31.3)
Region of Southern Denmark	3,445 (15.3)	3,171 (16.0)	274 (10.0)
Capital Region of Denmark	6,100 (27.1)	5,349 (27.1)	751 (27.5)
Region Zealand	4,348 (19.3)	3,772 (19.1)	576 (21.1)
**Cancer stage *N* (%)**			
Missing/Unknown	1,443 (6.4)	1,241 (6.3)	202 (7.4)
Stage I	4,873 (21.7)	3,734 (18.9)	1,139 (41.8)
Stage II	1,807 (8.0)	1,521 (7.7)	286 (10.5)
Stage III	4,089 (18.2)	3,678 (18.6)	411 (15.1)
Stage IV	10,280 (45.7)	9,591 (48.5)	689 (25.3)
**History of other cancer, *N* (%)**	6,312 (28.1)	5,435 (27.5)	877 (32.2)
**History of COPD, *N* (%)**	4,743 (21.1)	3,831 (19.4)	912 (33.4)
Lung cancer patient pathway referral, *N* (%)*	19,760 (87.9)	17,141 (86.7)	2,619 (96.0)
Low-dose chest CT scan (without contrast), *N* (%)*	9,692 (43.1)	7,481 (37.8)	2,211 (81.1)
Low-dose chest CT scans (without contrast), *N* (%)*			
0 scan	12,800 (56.9)	12,284 (62.2)	516 (18.9)
1 scan	5,652 (25.1)	5,122 (25.9)	530 (19.4)
2 scans	1,889 (8.4)	1,405 (7.1)	484 (17.7)
> 2 scans	2,151 (9.6)	954 (4.8)	1,197 (43.9)
Chest CT scans with contrast, *N* (%)*	19,852 (88.3)	17,230 (87.2)	2,622 (96.1)
Chest CT scans with contrast, *N* (%)*			
0 scan	2,640 (11.7)	2,535 (12.8)	105 (3.9)
1 scan	6,070 (27.0)	5,722 (29.0)	348 (12.8)
2 scans	6,522 (29.0)	5,980 (30.3)	542 (19.9)
> 2 scans	7,260 (32.3)	5,528 (28.0)	1,732 (63.5)
Any chest CT scan, *N* (%)*	21,162 (94.1)	18,454 (93.4)	2,708 (99.3)
Any chest CT scan, *N* (%)*			
0 scan	1,330 (5.9)	1,311 (6.6)	19 (0.7)
1 scan	5,831 (25.9)	5,651 (28.6)	180 (6.6)
2 scans	4,832 (21.5)	4,593 (23.2)	239 (8.8)
> 2 scans	10,499 (46.7)	8,210 (41.5)	2,289 (83.9)

The sampling of the cohort is described on Page 6 of the Supplemental Material.

* Within 5 years preceding a lung cancer diagnosis. Abbreviations: COPD: chronic obstructive pulmonary disease; CT: computed tomography.

## Discussion and conclusion

In this nationwide, population-based study of patients with a pulmonary nodule diagnosis, the incidence remained relatively stable from 2018 to 2022, although the number of chest CT scans increased slightly during the period. On average, 192.1 chest CT scans were performed for every 100 patients with a pulmonary nodule from 2018 to 2022, resulting in a total of 83,004 CT scans. This accounts for at least 5.2% of the 1.6 million chest CT scans conducted in Denmark during the same period. More than 30% lacked a follow-up chest CT scan 1 year after the nodule diagnosis. The risk of lung cancer was 3.6% 1 year after nodule diagnosis, while 7% had died within this period. For those being cancer-free and surviving until 12+1 months after the nodule diagnosis, the risk of lung cancer was low overall and below one percentage point for those who did not receive a chest CT scan.

Although pulmonary nodules are commonly encountered in clinical practice, few population-based studies have examined the occurrence of pulmonary nodules over time. The occurrence has mainly been examined among selected patients from lung cancer screening trials and hospital departments [5, 24–26]. Our results are comparable to those of a cohort study including all adults from the Kaiser Permanente Southern California healthcare system. They found an increase in pulmonary nodule incidence from 4.7 per 1,000 person-years in 2008 to 5.4 per 1,000 person-years in 2012, corresponding to a prevalence of 1.7% in the period [9]. While our study found a similar prevalence among Danish adults, the incidence in our study was lower at 1.94 cases per 1,000 person-years in 2022. In contrast, a US study using the Surveillance Epidemiology and End Results (SEER) Program-Medicare database reported a much higher pulmonary nodule diagnosis rate of 11.3 per 1,000 person-years among adults aged ≥ 65 years, both with and without cancer (a prevalence of 5%) [27]. This finding may align with estimates from our study among patients aged ≥ 65 years (SIR of 4.5 per 1,000 person-years in 2022; data not shown). Unlike existing studies, we focused on all Danish adults, irrespective of age and risk profile, using nodule diagnosis codes confirmed at respiratory or internal medicine hospital departments. Furthermore, differences in patient characteristics across cohorts and the varying size threshold used to define a nodule in clinical practice might also explain any discrepancies [5, 26]. Still, in our study, 12% of lung cancer patients had pulmonary nodules, which aligns with the 16% reported by SEER-Medicare [27].

Prior studies examining adherence to nodule follow-up were often small, included primarily selected patients, and showed considerable variation in adherence to follow-up depending on the setting, patient and nodule characteristics, and guideline awareness among radiologists and physicians [10–12]. While one study reported that only 29% of nodules detected in a hospital emergency department received a follow-up chest CT scan [10], other studies found guideline-concordant follow-up rates between 54 and 58% using hospital clinical pulmonary nodule databases, consistent with our findings [11, 12]. The low proportion receiving follow-up may be explained by nodules being small, thus not requiring follow-up, or by nonspecific lesions being deemed benign at time of diagnosis.

As some nodules may represent early-stage lung cancer [6], the consequences of missing guideline-recommended follow-up may have clinical implications. For example, a Danish study examined 4,066 patients with stage IV lung cancer and found that 552 patients (13.6%) had a chest CT scan within the 2 years before the diagnosis, with 245 of these having a nodule mentioned on their scan. The radiologist missed a nodule in 7.6% of the remaining 307 patients [28]. Overall, 36% of 245 patients with a nodule were inadequately monitored, and authors estimated that 2.5% of stage IV lung cancers in that cohort could have been detected earlier with recommended surveillance [28]. Clinical characteristics such as ethnicity, nodule size, history of COPD, and being seen by a pulmonologist may be associated with following the guidelines for single pulmonary nodules [11]. In our landmark analysis, we observed only slight differences in age, cancer history, and COPD between patients who had, versus those who had not, received at least one chest CT scan at 12+1 months after the nodule diagnosis. Still, those not receiving a chest CT scan had a lower lung cancer risk than those receiving a chest CT scan. This suggests that not receiving a follow-up CT scan was less likely due to oversight and more likely reflected clinical decisions based on stable nodule size on prior scans, benign morphology such as hamartoma and chondroma, nodules that were clearly deemed non-suspicious for malignancy, or nodules smaller than 5 mm, not requiring follow-up [3, 19].

We found that the risk of lung cancer after a nodule diagnosis at 12 and 24 months was consistent with the risk observed in the US SEER-Medicare cohort of 3.2 and 4.7%, respectively [27]. In contrast, data from Veterans Affairs hospitals showed a higher risk (9%) during an unspecified time [13]. Again, the setting and patient characteristics may explain the variation in risk, as, for example, increasing age, smoking history, and nodule size and location are associated with subsequent lung cancer diagnosis [29].

Our study had limitations. First, the diagnosis of pulmonary nodules has not been validated, and R91.9 may have been used for other pulmonary structures. Furthermore, we could not capture cases where pulmonary nodules were undetected or radiologically detected but not registered with a diagnosis code. To increase specificity at the expense of lower sensitivity, we included only patients diagnosed in internal medicine or pulmonary medicine departments, as diagnoses from other departments might reflect different lung abnormalities or nodules that do not require follow-up. Thus, we may have underestimated the occurrence of nodules. Second, the external validity of our results may be limited because R91.9 may not be used consistently across countries to identify pulmonary nodules. Third, the data structure of DNPR was updated in 2019, resulting in incomplete records, potentially explaining the slight decline in nodule incidence in 2019 [30]. Fourth, we had no information on the type, size, and appearance of nodules where part-solid and non-solid nodules more often represent lung cancer or precancerous lesions. We also lacked data on patients’ smoking status. As these features influence cancer risk and follow-up strategy, such information would enhance our understanding of why more than 30% of patients with pulmonary nodules do not receive a chest CT scan within 12 months after the diagnosis.

## Conclusion

The incidence of pulmonary nodules in Denmark remained relatively stable between 2018 and 2022, with approximately 8,600 new cases each year. Follow-up after a nodule diagnosis was relatively low, with only 68% of patients receiving a follow-up chest CT within 12 months. 3.6% of patients were subsequently diagnosed with lung cancer. Given the low incidence of lung cancer among those without a follow-up CT scan, the lack of follow-up was likely due to appropriate clinical evaluations considering nodule size, imaging characteristics, and patient-specific cancer risk factors.

## Supplementary Material



## Data Availability

The data are available for research upon request to the Danish Health Data Authority and within the framework of the Danish data protection legislation and any required permissions from authorities.
