# Supplementary material for: Pulmonary nodules in Denmark: occurrence, resource use, and risk of lung cancer and death
Source: Acta Oncol. 2025 Nov 2;64:44711. doi: 10.2340/1651-226X.2025.44711 (PMC12593931; doi:10.2340/1651-226X.2025.44711)
Supplement: Supplementary file 1 [file AO-64-44711-s1.pdf]

Supplementary material has been published as submitted. It has not been copyedited, or typeset by Acta Oncologica

Supplementary material

Title: Pulmonary nodules in Denmark: Occurrence, resource use, and risk of lung cancer and death

Content

Title	Pages
Supplemental Figure 1. Sampling of study population	2
Supplemental Table 1. ICD-10 diagnosis codes and administrative codes used in the study	3-4
Supplemental Figure 2. Study design illustrations	5
Pulmonary nodules among patients with lung cancer	6
Supplemental Table 2. Hospital at which pulmonary nodule diagnosis was recorded (2018—2022)	7
Supplemental Table 3. Descriptive characteristics of persons lung cancer free and surviving (landmark analysis)	8
Supplemental Figure 3. Risk of lung cancer in patients with pulmonary nodules (landmark analysis)	9
Supplemental Table 4. Risk of lung cancer in patients with pulmonary nodules (landmark analysis)	10
Supplemental Table 5. Total number and mean number per person per year of CT scans within 5 years before a lung cancer diagnosis	11

Supplemental Figure 1. Sampling of study population

All persons with a diagnosis of pulmonary nodules from a department of internal medicine or lung diseases (2018—2022)

n = 48,924

↓

Only first-time diagnoses

n = 44,669

↓

With a valid CPR number and age ≥18 years

n = 44,654

↓

Living in Denmark at time of diagnosis

n = 44,568

↓

With available information on region of residence

n = 44,555

↓

Without previous diagnosis of lung cancer

Final cohort. n = 43,209

↓

Landmark analysis. Alive and lung cancer-free at 12+1 months after pulmonary nodule diagnosis

n = 29,869

Supplemental Table 1. ICD-10 diagnosis codes and administrative codes used in the study

**Table S1. ICD-10 and administrative codes**				
	**Code (ICD-10 or administrative code)**	**IN/OUT/ED A/B/other**	**Comments**	**Registry**
**Pulmonary nodule**	R91.9	IN/OUT<br>A/B	To increase the positive predictive value, we focused on those diagnosed in internal or respiratory disease departments. These patients were identified by using records where the "speciale kode" was 01 (internal medicine) or 10 (pulmonary medicine) in LPR2 or where the "hovedspeciale" was categorized as "intern medicine" or "lungesygdomme" in LPR3.<br>The specific Danish hospitals were identified using the c_sgh variable in LPR2 and shak_sgh in LPR3.	DNPR
Cancer (excluding lung cancer)	C00-97 (not C33-C34)			DCR
**Lung cancer**	C33-34	n/a		DCR
Stage I	C33-34	n/a		DCR
Stage II	C33-34	n/a		DCR
Stage III	C33-34	n/a		DCR
Stage IV	C33-34 + C_tnm_m: AZCD41, AZCD41A, AZCD41B, AZCD41C	n/a		DCR
Unknown	C33-34 + C_tnm_m: AZCD49 (no information on distant metastases)	n/a		DCR
**Chest CT without contrast**	UXCC00, UXCC75 in any case without UXZ10* and without UXZ12	n/a	Chest CT without concurrent code for contrast<br>If any registration of UXCC+UXZ10, all registrations on the current day is ignored	DNPR
**Chest CT with contrast (including PET/CT)**	UXCC00, UXCC00A, UXCC70, UXCC75, UXCC77, UXCC (without any subcode), WDLBFXXXX, WMACPXYXX	n/a	UXC-codes were identified in combination with the codes: UXZ10 or UXZ12	DNPR
**Any chest CT scan**	The definitions used for *Chest CT without contrast* and *chest CT with contrast*			
**Lung cancer patient pathway referral**	AFB26A	n/a	If malignancy is suspected, the person is referred to further diagnostic evaluation through a standardized cancer patient pathway. These pathways were introduced in 2008 to obtain a timely diagnosis and ultimately improve the prognosis for cancer patients. Patients referred to a cancer patient pathway may either be	

			undergoing ongoing diagnostic tests (active investigations/in contact with the hospital) or have previously completed diagnostic cancer tests.	
**Other cancer patient pathway referral**	AFA01, AFBxxA, AFD01A	n/a	If malignancy is suspected, the person is referred to further diagnostic evaluation through a standardized cancer patient pathway. These pathways were introduced in 2008 to obtain a timely diagnosis and ultimately improve the prognosis for cancer patients. Patients referred to a cancer patient pathway may either be undergoing ongoing diagnostic tests (active investigations/in contact with the hospital) or have previously completed diagnostic cancer tests.	
**Chronic obstructive pulmonary disease**	DJ43-44	IN/OUT<br>A/B		DNPR

Supplemental Figure 2. Study design illustrations

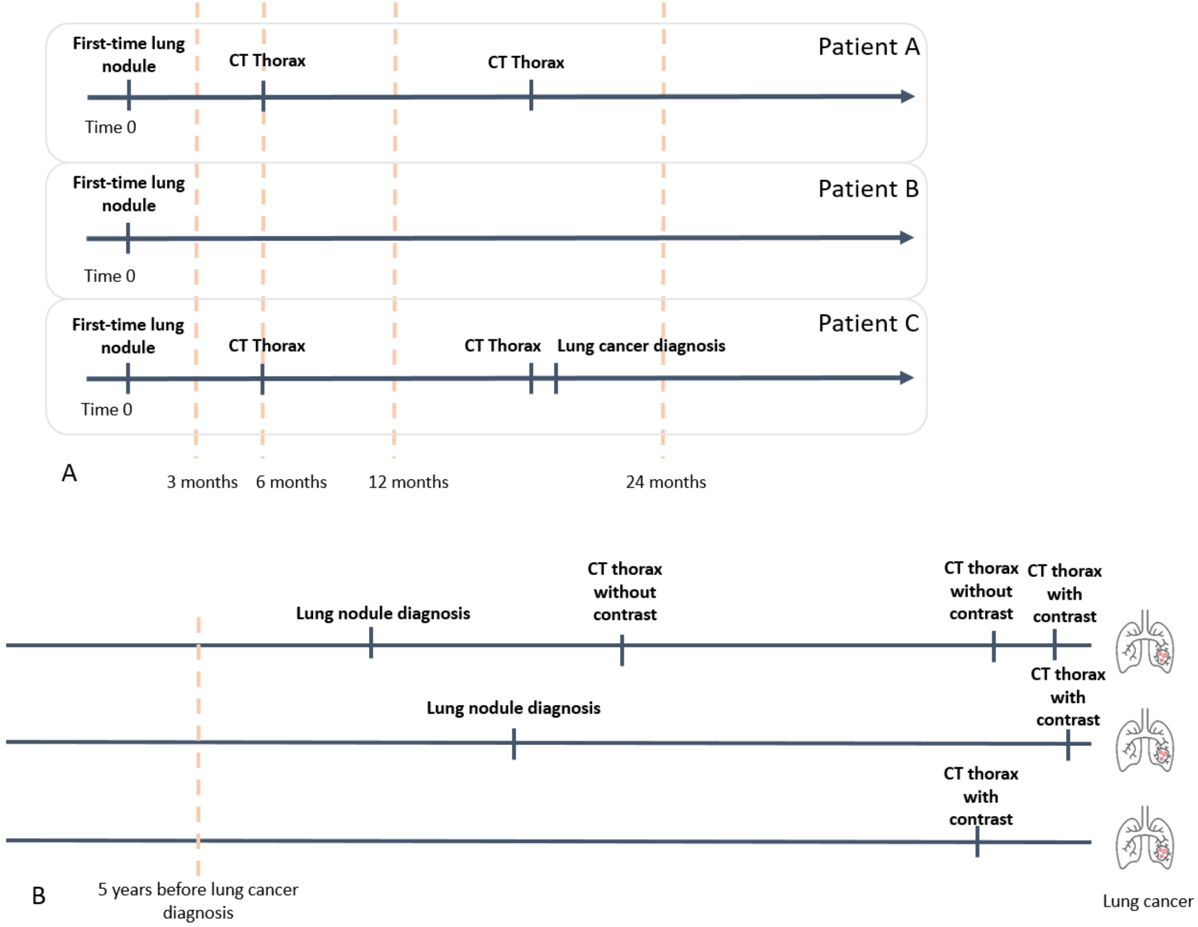

Panel A: Design of main analysis – cohort study following patients until lung cancer, emigration, death or end of study. Panel B: sensitivity analysis examining pulmonary nodules and CT use among patients with lung cancer.

Pulmonary nodules among patients with lung cancer

We conducted a cross-sectional study of all adults with a first-time diagnosis of lung cancer recorded in the Danish Cancer Registry during 2018-2022. The index date was the date of lung cancer diagnosis. Patients were required to have lived in Denmark in the five years preceding the index date. We assessed their characteristics and the number of chest CT scans within 5 years preceding the lung cancer diagnosis according to the presence of a previous pulmonary nodule diagnosis. We focused on distributions of age, sex, cancer stage, year of cancer diagnosis, lung cancer patient pathway referrals, Danish regions, and hospital departments where the cancer diagnosis was given.

Supplemental Table 2. Hospital at which pulmonary nodule diagnosis was recorded (2018—2022)

	N (%)
**Total**	
**Hospital**	
Aalborg sygehus	1,891 (4.4)
Aalborg universitetshospital, Thisted	85 (0.2)
Aarhus Universitethospital	2,105 (4.9)
Amager og Hvidovre Hospital	1,017 (2.5)
Bispebjerg	5,660 (13.3)
Bornholms Centralsygehus	32 (0.1)
CPH Privathospital A/S	<5
Esbjerg Centralsygehus	1,753 (4.1)
Frederica og Kolding Sygehuse	30 (0.1)
Frederiksborg Amts Sundhedsvæsen	942 (2.2)
Horsens sygehus	968 (2.3)
Herlev-Gentofte Hospital	6,560 (15.4)
Hjørring-Brønderslev Sygehus	221 (0.5)
Hospitalsenhed Midt	4,405 (10.3)
Hospitalsenheden Vest	1,691 (4.0)
Medicinsk Speciallægeklinik	<5
Odense Universitetshospital	2,704 (6.3)
Randers Centralsyghus	2,613 (6.1)
Region Sjællands Sygehusvæsen	6,916 (16.2)
Regionshospitalet Gødstrup	259 (0.6)
Rigshospitalet	410 (1.0)
Sygehus Sønderjylland	2,080 (4.9)
Vejle Sygehus	259 (0.6)
Missing	1,897 (4.4)

Supplemental Table 3. Descriptive characteristics of patients with lung cancer-free survival 12+1 months after a pulmonary nodule diagnosis, according to history of a chest CT scan

	**Received a chest CT scan N (%)**	**No chest CT scan N (%)**
**Total**	21,032	8,837
**Follow-up time in years (median, IQR)**	1.8 (0.8;2.9)	1.8 (0.9;2.8)
**Sex**		
Men	11,091 (52.7)	4,640 (52.5)
Women	9,941 (47.3)	4,197 (47.5)
**Age in years (median, IQR)**	70.0 (60.8; 77.0)	67.7 (56.2; 76.3)
Age <60 years	4,964 (23.6)	2,863 (32.4)
Age 60-70 years	5,565 (26.5)	2,067 (23.4)
Age 70-80 years	7,287 (34.6)	2,538 (28.7)
Age >80 years	3,216 (15.3)	1,369 (15.5)
**Year**		
2018	-	-
2019	5,732 (27.3)	2,018 (22.8)
2020	4,689 (22.3)	2,298 (26.0)
2021	5,107 (24.3)	2,318 (26.2)
2022	5,504 (26.2)	2,203 (24.9)
**Comorbidities**		
COPD	3,966 (18.9)	1,363 (15.4)
Cancer (excl. lung cancer)	6,571 (31.2)	1,796 (20.3)
**History of referral to cancer patient pathways**		
Lung cancer patient pathway within 6 months prior to index date*	475 (2.3)	<5
Other cancer patient pathway within 6 months prior to index date*	920 (4.4)	166 (1.9)

*Index date not included

Supplemental Figure 1 presents the sampling of the cohort.

The history of any chest CT scan was assessed 12+1 months before the landmark.

Supplemental Figure 3. Unadjusted risk of lung cancer in patients with lung cancer-free survival 12+1 months after a pulmonary nodule diagnosis, according to history of a chest CT scan

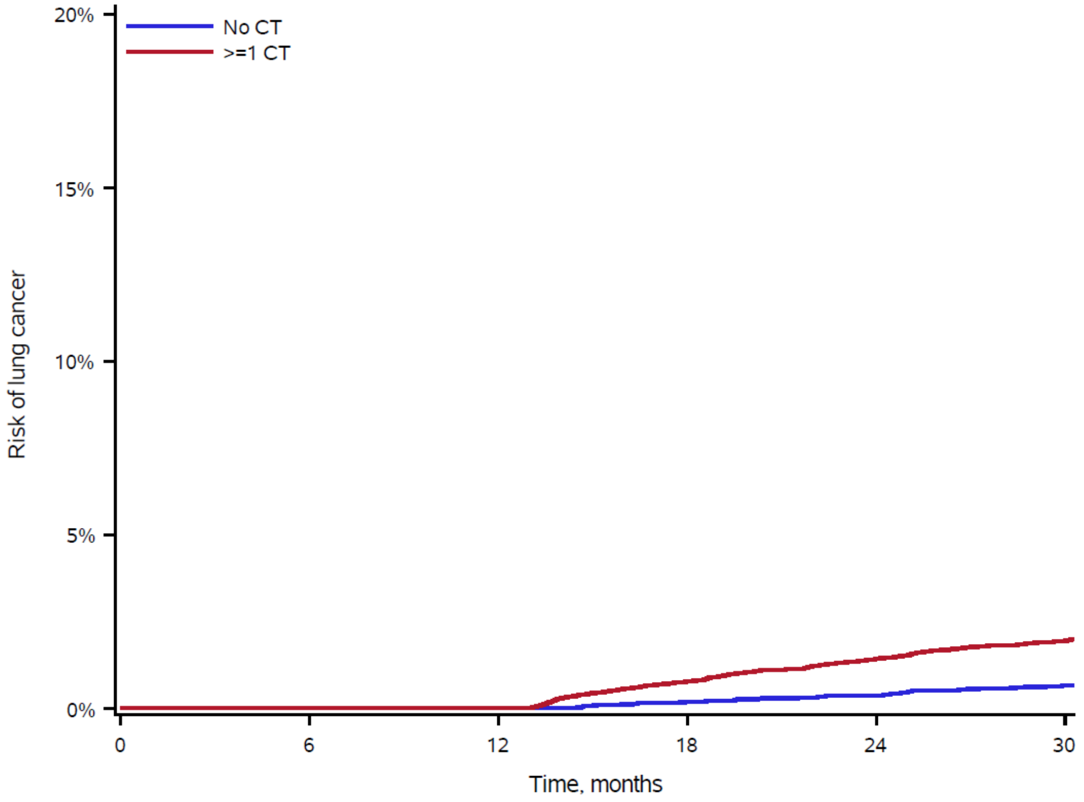

Supplemental Figure 1 presents the sampling of the cohort. The history of any chest CT scan was assessed 12+1 months before the landmark.

Supplemental Table 4. Risk of lung cancer in patients with lung cancer-free survival 12+1 months after a pulmonary nodule diagnosis, according to history of a chest CT scan

**Outcome**	**Group**	**Follow-up**	**Cumulative incidence % (95% CI)**
**Lung cancer**	**With CT**	13-18 months	0.8 (0.7; 0.9)
		13-24 months	1.4 (1.3; 1.6)
	**Without CT**	13-18 months	0.2 (0.1; 0.3)
		13-24 months	0.4 (0.3; 0.5)

Supplemental Figure 1 presents the sampling of the cohort. The history of any chest CT scan was assessed 12+1 months before the landmark.

Supplemental Table 5. Total number and mean number per person per year of CT scans within 5 years before a lung cancer diagnosis, according to the history of a pulmonary nodule diagnosis

Chest CT modularity	Low-dose chest CT scan (without contrast) (mean number per person per year)		Chest CT scan (with contrast) (mean number per person per year)		Any chest CT scan (mean number per person per year)	
**Pulmonary nodule diagnosis within 5 years**	**No**	**Yes**	**No**	**Yes**	**No**	**Yes**
**Total**	11,761 (0.12)	7,216 (0.53)	39,676 (0.40)	9,731 (0.71)	51,437 (0.52)	16,947 (1.24)
**Sex**						
Women	6,085 (0.12)	4,062 (0.56)	20,102 (0.40)	5,036 (0.69)	26,187 (0.52)	9,098 (1.25)
Men	5,676 (0.12)	3,154 (0.50)	19,574 (0.41)	4,695 (0.74)	25,250 (0.53)	7,849 (1.24)
**Age**						
<60	1,001 (0.09)	582 (0.51)	4,204 (0.37)	759 (0.66)	5,205 (0.46)	1,341 (1.17)
60-70	3,167 (0.11)	1,867 (0.51)	11,382 (0.40)	2,593 (0.71)	14,549 (0.52)	4,460 (1.23)
70-80	5,453 (0.13)	3,446 (0.55)	17,432 (0.42)	4,694 (0.75)	22,885 (0.56)	8,140 (1.29)
≥80	2,140 (0.12)	1,321 (0.52)	6,658 (0.37)	1,685 (0.66)	8,798 (0.48)	3,006 (1.17)
**Year of diagnosis**						
2018	1,616 (0.08)	936 (0.38)	5,121 (0.27)	1,252 (0.51)	6,737 (0.35)	2,188 (0.89)
2019	2,376 (0.12)	1,269 (0.53)	7,953 (0.41)	1,722 (0.72)	10,329 (0.53)	2,991 (1.25)
2020	2,423 (0.12)	1,623 (0.55)	8,569 (0.43)	2,154 (0.73)	10,992 (0.55)	3,777 (1.28)
2021	2,522 (0.13)	1,672 (0.58)	8,955 (0.44)	2,282 (0.79)	11,477 (0.57)	3,954 (1.36)
2022	2,824 (0.14)	1,716 (0.58)	9,078 (0.45)	2,321 (0.79)	11,902 (0.59)	4,037 (1.37)
**Cancer stage**						
Missing/Unknown	532 (0.09)	377 (0.37)	2,186 (0.35)	595 (0.59)	2,718 (0.44)	972 (0.96)
Stage I	4,732 (0.25)	3,843 (0.67)	10,453 (0.56)	4,526 (0.79)	15,185 (0.81)	8,369 (1.47)
Stage II	1,298 (0.17)	804 (0.56)	3,542 (0.47)	1,040 (0.73)	4,840 (0.64)	1,844 (1.29)
Stage III	1,749 (0.10)	933 (0.45)	7,590 (0.41)	1,477 (0.72)	9,339 (0.51)	2,410 (1.17)
StageIV	3,450 (0.07)	1,259 (0.37)	15,905 (0.33)	2,093 (0.61)	19,355 (0.40)	3,352 (0.97)
**Region**						
North Denmark Region	1,283 (0.09)	470 (0.34)	4,171 (0.30)	912 (0.67)	5,454 (0.39)	1,382 (1.01)
Central Denmark Region	4,296 (0.18)	2,777 (0.65)	10,560 (0.45)	3,061 (0.72)	14,856 (0.63)	5,838 (1.37)
Region of Southern Denmark	1,477 (0.09)	606 (0.44)	5,471 (0.35)	823 (0.60)	6,948 (0.44)	1,429 (1.04)
Capital Region of Denmark	2,772 (0.10)	2,008 (0.53)	13,165 (0.49)	3,058 (0.81)	15,937 (0.60)	5,066 (1.35)
Region Zealand	1,933 (0.10)	1,355 (0.47)	6,309 (0.33)	1,877 (0.65)	8,242 (0.44)	3,232 (1.12)
**History of cancer**						
No	7,465 (0.10)	4,715 (0.51)	25,398 (0.35)	5,921 (0.64)	32,863 (0.46)	10,636 (1.15)
Yes	4,296 (0.16)	2,501 (0.57)	14,278 (0.53)	3,810 (0.87)	18,574 (0.68)	6,311 (1.44)
**History of COPD**						
No	8,993 (0.11)	4,729 (0.52)	31,004 (0.39)	6,165 (0.68)	39,997 (0.50)	10,894 (1.20)
Yes	2,768 (0.14)	2,487 (0.55)	8,672 (0.45)	3,566 (0.78)	11,440 (0.60)	6,053 (1.33)

The sampling of the cohort is described on Page 6 of the Supplemental Material. Abbreviations: COPD, chronic obstructive pulmonary disease; CT, computed tomography
